# MicroRNA-122 overexpression suppresses the colon cancer cell proliferation by downregulating the astrocyte elevated gene-1/metadherin oncoprotein

**DOI:** 10.1080/07853890.2025.2478311

**Published:** 2025-04-10

**Authors:** Sarubala Malayaperumal, Sushmitha Sriramulu, Ganesan Jothimani, Antara Banerjee, Hong Zhang, Shabana Thabassum Mohammed Rafi, Ilangovan Ramachandran, Rajesh Kanna NR, Xiao-Feng Sun, Surajit Pathak

**Affiliations:** ^a^ Faculty of Allied Health Sciences, Chettinad Academy of Research and Education, Chettinad Hospital and Research Institute, Chennai, India; ^b^ Department of Medical Sciences, School of Medicine, Orebro University, Örebro, Sweden; ^c^ Department of Endocrinology, Dr. ALM PG Institute of Basic Medical Sciences, University of Madras, Taramani Campus, Chennai, India; ^d^ Department of Pathology, Chettinad Academy of Research and Education, Chettinad Hospital and Research Institute, Chennai, India; ^e^ Division of Oncology, Department of Biomedical and Clinical Sciences, Linköping University, Linköping, Sweden

**Keywords:** miR-122, colon cancer, inflammatory cytokines, apoptosis, TCGA-COAD, AEG-1/MTDH

## Abstract

**Background:**

MicroRNAs (miRNAs) are small non-coding RNAs that regulate essential cellular functions, such as cell adhesion, proliferation, migration, invasion, and programmed cell death, and therefore, alterations in miRNAs can contribute to carcinogenesis. Previous studies have shown that miRNA-122 is abundant in the liver and regulates cell proliferation, migration, and apoptosis. However, the expression pattern and mechanism of actions of miR-122 remain primarily unknown in colon cancer.

**Methods:**

In this study, we analyzed The Cancer Genome Atlas Colon Adenocarcinoma (TCGA-COAD) database to assess the clinical significance of astrocyte elevated gene-1 (AEG-1)/metadherin (MTDH) and miR-122 in colon cancer. MiR-122 overexpression studies were performed in HCT116, SW480, and SW620 cell lines. Dual-luciferase assay was carried out to confirm the interaction between AEG-1 and miR-122. *In vivo*-JetPEI–transfection reagent was used for *in-vivo* transient transfection of miR-122 in the AOM/DSS-induced colon tumor mouse model.

**Results:**

Our results demonstrate that miR-122 was downregulated in colon cancer cells, and it influences the expressions of apoptotic factors and inflammatory cytokines. MiR-122 overexpression in HCT116, SW480, and SW620 cells showed upregulation of *Caspase 3*, *Caspase* *9*, and *BAX* and decreased expression of *BCL2*, which are pro-apoptotic and anti-apoptotic members that maintain a ratio between cellular survival and cell death. *In vivo* transient transfection of miR-122 mimic in AOM/DSS induced colon tumor mouse model showed less inflammation and disease activity. The TCGA-COAD data indicated that AEG-1 expression was higher in patients with low expression of miR-122 and lower AEG-1 expression in patients with higher expression miR-122.

**Conclusion:**

Our findings highlight the key role of miR-122 in the high grade of colonic inflammation, and possibly in colon cancer, and the use of miR-122 mimic might be a therapeutic option.

## 1. Introduction

Colon cancer is the 3^rd^ most common cause of cancer globally, with the survival rate ranging from 50 to 93% depending on the cancer stage [1]. As early diagnosis is essential for effective treatment in patients, pathological examination and colonoscopy remain the gold-standard methods to screen colorectal cancer. Many invasive and non-invasive methods, including blood and faecal examinations, are available for the early detection of colon cancer [2]. Despite all the advancements, the mortality rate increases annually, and a better understanding of underlying molecular mechanisms is needed to find new biomarkers for effective screening and treatment of colon cancer [3]. MicroRNAs (miRNAs/miRs) are small non-coding ribonucleotides that play a crucial role in the cellular proliferation, migration, and apoptosis [2]. These miRNAs bind to the target mRNA through the seed region, which then leads to mRNA cleavage and translational repression [4–6]. Some miRNAs act as tumour suppressors, whereas some function as oncogenes, i.e. oncomiRs. MicroRNA-122 is the most abundant miRNA in the human liver, which accounts for a total of 70% of the miRNA population [7], and plays a vital role in cholesterol and fatty acid metabolism. Studies have reported that miR-122 is downregulated in hepatocellular carcinoma (HCC), and its overexpression leads to the inhibition of proliferation, invasion, and apoptosis of HCC cells [8]. Recently, numerous studies have focused on miR-122 expression in various cancers, suggesting it as one of the potential therapeutic targets. However, there are only few studies on the role of miR-122 in colorectal cancer (CRC). Moreover, there are no studies on the impact of the therapeutic potential of miR-122 in colorectal cancer progression. Our previous study demonstrated that miRNA-122 reduces cellular proliferation and migration while inducing the apoptosis of colon cancer cells [9]. The present study was designed to determine the target genes of miR-122 that mediate the biological effects in colon cancer cells using *in vitro* and *in vivo* models. Bioinformatics tools were used to identify the top interactors of miR-122, followed by scrutinizing the astrocyte elevated gene-1 (AEG-1)/metadherin (MTDH). We downloaded *miR-122* and AEG-1/MTDH expression data from TCGA-COAD databases, and we found an association between *miR-122* and AEG-1/MTDH expression.

## 2. Materials and methods

### 2.1. Cell culture and expansion

SW480, SW620, and HCT-116 cell lines were purchased from National Centre for Cell Science (NCCS), Pune. All the cell lines were mycoplasma free. The cells were cultured in Dulbecco’s Modified Eagle’s Medium (DMEM) (Catalogue #11885084, Gibco) with 10% fetal bovine serum (FBS) (Catalogue # 26140087, Gibco), 1X Penicillin/Streptomycin (Catalogue # 15140122, Gibco) and incubated in 5% CO_2_ at 37 °C.

### 2.2. Screening by miScript miRNA PCR array and functional enrichment

Total RNA was extracted from HCT-116 cells using TRIzol reagent (Catalogue # 15596026, Invitrogen, USA) according to the manufacturer’s protocol. The concentration of the RNA was quantified using NanoDrop spectrophotometer and cDNA was synthesized with miScript II RT kit and subjected to miRNA expression profiling using Human Cancer Pathway Finder miScript miRNA PCR Array (Qiagen Catalogue # MIHS-102ZA). miRnet online tool was used to enrich the miRNA family function, Reactome analysis, and molecular biology function analysis of the miRNAs.

### 2.3. Overexpression of miRNA-122

The precursor for miRNA-122 (Catalogue # AM17100) (mature miRNA sequence UG-GAGUGUGACAAUGGUUUG) miRNA-122 mimic (Catalogue # 4464066) was purchased from Ambion^®^ Life Technologies. Using Lipofectamine RNAiMAX (Catalogue # 13778075, Invitrogen, Thermo Fisher Scientific), miRNA-122 precursors was transfected into HCT-116, SW480, and SW620 cells following the manufacturer’s instructions. The transfected cells and cell supernatants were collected for further functional characterization.

### 2.4. Quantification of VEGF, IL-6, and cytochrome C

Enzyme-linked immunosorbent assay (ELISA) was performed to detect the levels of pro-inflammatory cytokines, such as vascular endothelial growth factor (VEGF) (Catalogue # E0080Hu, Bioassay Technology), Interleukin-6 (IL-6) (Catalogue # E0226Hu, Bioassay Technology) and apoptotic marker Cytochrome C (Catalogue # KTE62179, Abbkine). The supernatants were collected to determine the levels of pro-inflammatory cytokines and Cytochrome C using the Bioassay ELISA kits according to the manufacturer’s protocol. The OD at 450 nm was measured using an ELISA plate reader (Robonik Readwell touch Microplate Analyzer, India).

### 2.5. Target prediction and dual-luciferase assay

The list of potential miR-122 interactors, which included genes and transcription factors as well as their direct interactors, was screened and enriched using STRING entries from miRNet 2.0. The compiled gene set of miR-122 major interactors was affirmed further using published research [10] (data reported in our previous study). The *AEG-1*/*MTDH* gene was scrutinized with its binding score and due to the highest mutational rate in colon cancer determined with the TNM plot database. The online bioinformatics tool TargetScan was used to predict the target binding sequence of miR-122 with *AEG-1*/*MTDH*. The 3′UTR region of *AEG-1/MTDH* was cloned into the pmiRGlo vector, amplified, and labelled as pmiRGLO-MTDH-WT, and the mutant type was labelled as pmiRGLO-MTDH-Mut. In brief, the 3′UTR *AEG-1/MTDH* sequence of wild type and mutant were commercially synthesized with Not1 restriction site internal sequence (Table 1). The pmiR-GLO vector is about 7.3 kb with multiple cloning sites (MCS), ampicillin resistance gene (ampr), and hRluc-neo gene (renilla luciferase). PmiR-GLO vector was restriction digested using SacI and XbaI enzymes, which created sticky ends in the plasmid. Then, the synthesized strands were annealed and ligated into the digested plasmid with T4 DNA ligase. The ligated recombinant plasmid was then transformed into *E. coli* DH5α strain and plated on nutrient agar plates containing ampicillin (Amp+). The colonies from amp + were selected for plasmid DNA isolation and screened for the presence of the plasmid with gene of interest (GOI) by restriction digestion of NotI internal sequence. The purified GOI+ plasmids and miR-122 mimic were then co-transfected into HCT-116 cells and incubated for 24 h at standard conditions. After 48 h of transfection, a dual-luciferase reporter assay system was used to analyze the luciferase activity, and luminescence was measured using the Promega GLO-max system. Luciferase activity in wild-type *AEG-1/MTDH* 3′ UTR was compared to the control, and each assay was performed in triplicate.

**Table 1.** Sequence of the 3′UTR region of AEG-1/MTDH DNA fragments.

AEG-1/MTDH WT	5′GCGGCCGCAAGATCGTGCCACTGCACTCCAT3′
	5′ATGGAGTGCAGTGGCACGATCTTGCGGCCGC3′
AEG-1/MTDH mut	5′GCGGCCGCAAGATCGTGCCACTGCAGGCAT3′
	5′ATGCCTGCAGTGGCACGATCTTGCGGCCGC3′

### 2.6. Immunofluorescence analysis

Control and *miR-122* overexpressing cells were cultured in 24 well plates at the density of 1 x 10^5^ cells/per well. The cultured cells were washed with phosphate-buffered saline (PBS) and fixed with methanol for at least 10 min. Permeabilization of cells was carried out with 0.1% Triton-X 100 and blocked with bovine serum albumin (BSA) (Catalogue # 96409, SRL) for 30 min. The cells were then incubated overnight with AEG-1/MTDH antibody (Catalogue # 40-6400, Invitrogen, Thermo Fisher Scientific) raised in rabbits and developed with anti-rabbit Alexa Fluor antibody (Catalogue # A-21070, Invitrogen, Thermo Fisher Scientific). DAPI (Catalogue # D9542, Sigma Aldrich) was used to counterstain nucleic acid (Invitrogen, USA). The fluorescent images were observed and photographed using a fluorescent microscope (EVOS FLoid,Thermo Fisher Scientific). Quantitative analysis was done with ImageJ software.

### 2.7. Clinical characteristics of patients from The Cancer Genome Atlas analysis (Online database)

The *miRNA-122* and *AEG-1/MTDH* expression data for colon cancer were obtained from the TCGA GDC portal-https://portal.gdc.cancer.gov/ as of 11-10-2022). A total of 93 cases from TCGA and 31 cases from CPTAC containing miRNA expression data were downloaded. All the sample types containing miRNA expressions were found to be cancerous groups. As the data were sourced from TCGA public databases, and processed in compliance with TCGA publication criteria (https://cancergenome.nih.gov/publications/guidelines)), the Institutional Review Board of Chettinad Academy of Research and Education confirmed that ethical approval is not required as the data sets generated were in compliance with TCGA policies for data acess via the online database. Samples were sorted based on the *miR-122* reads per kilo-base million (RPKM), and outliers were removed based on the miRNA expression. The samples having an RPKM value of *miR-122* less than the median RPKM were categorized in the low miR-122 expression, and higher than the median RPKM was categorized in the high *miR-122* expression. The FPKMUQ value for *AEG-1/MTDH* was obtained from the available data. The differential expression of *AEG-1/MTDH* was studied by calculating the log10 of FPKMUQ value between the *miR-122* highly expressed and less expressed colon cancer patient tissues to identify their interplay.

### 2.8. Animal model

Male BALB/C mice (6–8 weeks old, weight 20–22 g) were purchased from Tamil Nadu Veterinary and Animal Sciences University (TANUVAS), Chennai, India. After receiving approval from the Chettinad Academy of Research and Education’s Institutional Animal Ethics Committee, all experiments were carried out (CARE) (IAEC1/Proposal:48/A. Lr:31/Dt:20.03.2020) according to the rules set forth by the Committee for the Purpose of Control and Supervision of Animal Experiments (CPCSEA).

All animals were received and handled with appropriate care. Mice were housed in pathogen-free animal facilities at Chettinad Academy of Research and Education, Chennai, India. They were kept at a constant 20–25 °C temperature, 50–70% humidity, and a 12-h light/dark cycle. All mice were provided with food and water *ad libitum*.

### 2.9. AOM/DSS-induced tumor mouse model

Azoxymethane (AOM) (Catalogue # A5486, Sigma Aldrich) and dextran sulfate sodium (DSS) (Catalogue # 99629, SRL) were used to induce colon tumor in mice. Briefly, AOM was administered intraperitoneally (i.p.) to mice at a dose of 10 mg/kg body weight. Two days following the AOM injection, 2% DSS was infused into drinking water for 7 continuous days, followed by 3 days of regular water. This treatment cycle was then repeated twice. The animals were grouped into 1) Healthy control (*n* = 5); 2) AOM/DSS-tumor induced mice group (*n* = 5); 3) AOM/DSS+miR-122 mimic treated mice group (*n* = 5).

### 2.10. In vivo *miR-122 transfection in mice*

MiR-122 mimic (3 µg) was diluted in 50 µL of 10% glucose solution and made up to 100 µL with sterile water, making the final glucose concentration 5%. AOM/DSS treated mice were injected intraperitoneally with miRNA-122 mimic (3 μg) using JetPEI *in vivo* transfection (Catalogue # 201-10G, Polypus transfection) reagent twice a week throughout the period till euthanasia. The disease activity index (DAI) was calculated by scoring the severity of the disease by recording parameters including body weight, food intake, and stool consistency on a weekly basis [11]. Following euthanasia, the colon was removed and rinsed with PBS, and tissues were collected for further studies, such as gene expression and immunohistochemistry.

### 2.11. Quantitative real-time RT-PCR

Total RNA was extracted from cancer cells and colon tissues using TRIzol reagent (Catalogue # 15596026 Invitrogen, USA) in accordance with the manufacturer’s protocol. The concentration was determined using NanoDrop spectrophotometer and reverse transcribed into cDNA using RT cDNA conversion kit (Eurogentech, RTCK-03). The miRNA-122 expression was quantified using a TaqMan probe, whereas the mRNA expression levels of *AEG-1/MTDH*, *BCL-2*, *BAX*, *Caspase-3*, and *Caspase-9* were detected using Real-time PCR SYBR green assay (Catalogue # UF-RSMT-B0701 Eurogentech) using β-actin as an endogenous control. The list of primers used are mentioned in Table 2.

**Table 2.** Mouse primer sequences for the gene of interest.

*β-actin*	CTGTCCCTGTATGCCTCTG ATGTCACGCACGATTTCC
*AEG-1/MTDH*	ATAACTCTCACACACAGGAC GCTTTTGAGGTATTCACTGG
*BAX*	GTGCCGGAACTGATCAGAAC CCAAAGTAGGAGAGGAGGCC
*BCL-2*	GCCTTCTTTGAGTTCGGTGG GAAATCAAACAGAGGCCGCA
*Caspase-3*	ATGGTTTGAGCCTGAGCAGA GGCAGCATCATCCACACATAC
*Caspase-9*	GCAGGCTCTGGATCTCGGC GCTGCTTGCCTGTTAGTTCGC

### 2.12. Immunohistochemistry

The colon tissues collected from control and treatment mice were formalin fixed. The tissues were paraffin-embedded and sectioned for immunohistochemical staining. Briefly, sections were deparaffinized, followed by heat-mediated antigen retrieval and permeabilization with Triton-X 100 (0.5%). Antigen-retrieved tissues were blocked with BSA and incubated with AEG-1/MTDH antibody (rabbit polyclonal) overnight and HRP-conjugated anti-rabbit antibody for 1 h. 3,3-Diaminobenzidine (DAB) was used as a substrate for HRP, and the pathological score was calculated by measuring the intensity of the color using ImageJ software.

### 2.13. Statistical analysis

All experiments were performed in triplicate, and a two-tailed unpaired student *t*-test with independent variables, and one-way ANOVA/multiple *t*-tests were performed using Graph Pad Prism V9. The data were considered statistically significant with *p* ≤ 0.05 and represented as asterisks * (*p*  ≤ 0.05), ** (*p* ≤ 0.01), and *** (*p* ≤ 0.001).

## 3. Results

### 3.1. Screening of miRNA in colon cancer cells and gene ontology analysis

**Figure 1.**
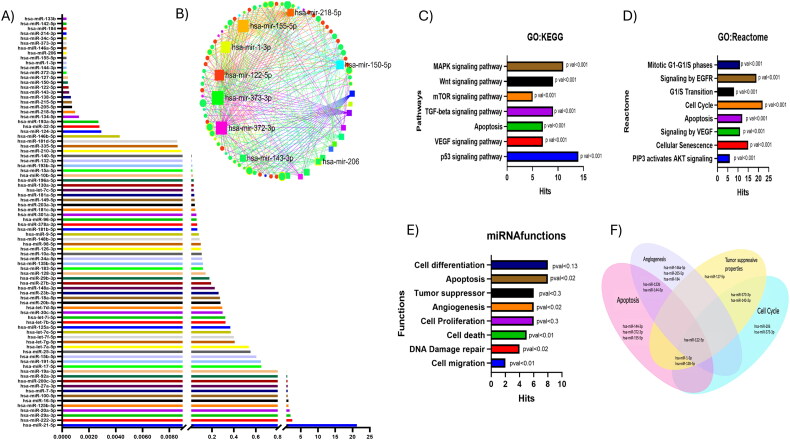
(A) The human cancer pathway Finder miScript miRNA PCR array-based profiling in colon cancer cells. (B) miRNA-mRNA interaction network of downregulated miRNAs: functional enrichment analysis of top 20 downregulated miRNAs. (C) GO: KEGG pathway analysis depicting major pathways of shortlisted miRNAs. (D) GO: Reactome analysis of miRNAs. (E) Major functions of downregulated miRNAs. (F) Venn diagram of miRNA based on miRNA functions.

The human cancer pathway finder miScript miRNA PCR array was used to profile the differentially expressed miRNAs in HCT-116 cells. The expression of 80 miRNAs involved in cancer signaling pathways was analyzed (Figure 1). The top 20 downregulated miRNAs were selected for gene ontology analysis and functional enrichment. All the downregulated miRNAs were found to be modulators of the cell cycle and seemed to be involved in functions like proliferation, migration, angiogenesis, and apoptosis. miR-133b, miR-142-5p, miR-184, miR-214-3p, miR-34c-5p, miR-373-3p, miR-146a-5p, miR-206, miR-155-5p, miR-1-3p, miR-144-3p, miR-372-3p, miR-127-5p, miR-150-5p, miR-122-5p, miR-143-3p, miR-138-5p, and miR-215-5p were downregulated whereas the expression of miR-92a-3p, miR-200c-3p, miR-27a-3p, miR-7-5p, miR-100-5p, miR-16-5p, miR-125b-5p, miR-20a-5p, miR-29a-3p, miR-222-3p, miR-21-5p was upregulated in the colon cancer cell line.

Functional enrichment analysis was performed on the top 20 downregulated miRNAs. miRNA-mRNA interactions network of miRNA revealed that each miRNA interacts with a minimum of 10 nodes (mRNA) represented in Figure 1B. KEGG and Reactome pathway analyses showed that these miRNAs were involved in most of cancer signalling pathways and cell cycle transitions (Figures 1C,D). miRNA function analysis revealed that most of these miRNAs are involved in important functions, such as cell proliferation, apoptosis, migration, and DNA damage repair mechanism, and few also possess tumour suppressive properties (Figure 1E). The enrichment of selected miRNAs revealed that miR-122 is involved in angiogenesis, programmed cell death and cell cycle regulation, and tumour suppressive properties, and hence it was taken for further investigation (Figure 1F).

### 3.2. miR-122 modulates key proteins that regulate programmed cell death and invasion of colon cancer cells

**Figure 2.**
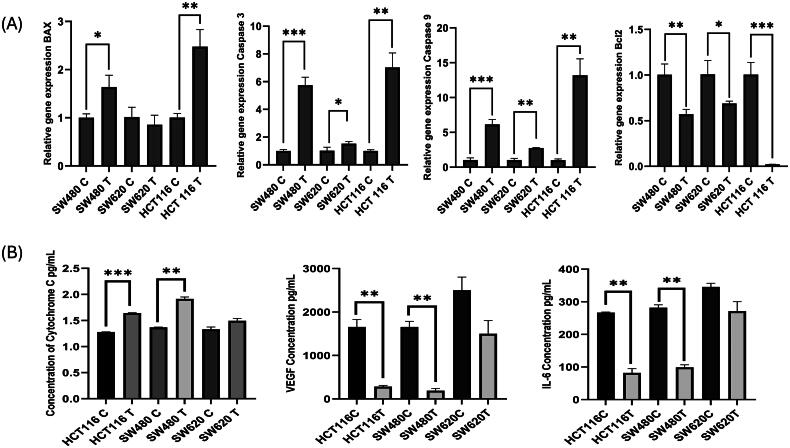
(A) Relative expression of *BAX*, *Caspase-3*, *Caspase-9* and BCL-2 in control and *miR-122* mimic transfected colon cancer cells. (B) Determination of mitochondrial protein cytochrome C and inflammatory cytokine levels in control and miR-122 overexpressed colon cancer cells (C: control; T: miR-122 mimic treated).

The mRNA levels of apoptotic genes were assessed by qRT-PCR in control and miR-122 overexpressed cells to determine if miR-122 restoration had any effect on apoptotic genes. MiR-122 mimic overexpression was shown to enhance the expression of *BAX* in colon cancer cells (HCT-116, *p* = 0.002; SW480, *p* = 0.01; SW620, *p* = 0.3), *Caspase-3* (HCT-116, *p* = 0.006; SW480, *p* = 0.0006; SW620, *p* = 0.03), and *Caspase-9* (HCT-116, *p* =0.0009; SW480, *p* = 0.0003; SW620, *p* = 0.003), which has a vital role in inducing programmed cell death and inhibit the expression of BCL-2, which is an anti-apoptotic factor (Figure 2A). Moreover, the mitochondrial protein level of cytochrome C was measured in control and transfected cells by ELISA (Figure 2B). The results revealed that cytochrome C level was significantly increased in miR-122 mimic transfected cells (HCT-116, *p* = 0.001; SW480, *p* = 0.0026; SW620, *p* = 0.06), indicating that miR-122 plays a crucial role in driving the programmed cell death. Our overall results suggest the miR-122 mimic overexpression increases the apoptotic rate and decreases the proliferation more effectively in the primary colon cancer cells.

### 3.3. miR-122 inhibits angiogenesis by regulating the secretion of inflammatory cytokines

Invasion and angiogenesis in tumor cells are closely associated with inflammatory cytokines like VEGF and IL-6. These inflammatory cytokines, which play a vital role in cell division and invasion were found to be very high in colon cancer. Following the transfection of *miR-122*, the expression level of *VEGF* was reduced significantly in colon cancer cells with a *p*-value = 0.0081 for HCT116, *p* ≤0.0045 for SW480, *p* ≤0.08 for SW620 and IL-6 production was decreased with a *p*-value 0.0025 for HCT116, *p* ≤ 0.0020 for SW480, *p* ≤ 0.07 for SW620 (Figure 2B).

### 3.4. Interaction between AEG-1 and miR-122

**Figure 3.**
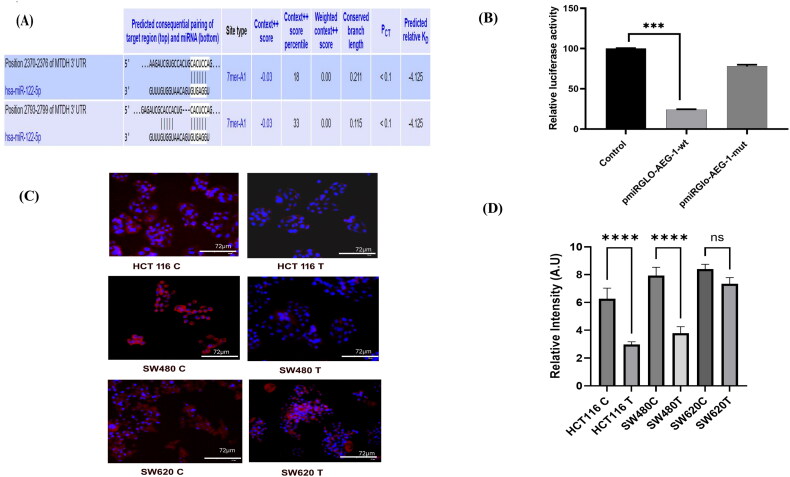
(A) Prediction of consequential pairing sequence through bioinformatics tool Targetscan. (B) Relative luciferase activity of pmiRGLO-MTDH-wt and pmiRGLO-MTDH-Mut. (C) Immunofluorescence analysis of AEG-1/MTDH protein in control and miR-122 transfected colon cancer cell lines. (D) Quantification analysis of immunofluorescence by ImageJ (expressed in arbitrary units).

Figure 3A shows the prediction of consequential pairing sequence through the bioinformatics tool Targetscan, and Figure 3B represents the relative luciferase activity of pmiRGLO-AEG-1-wt and pmiRGLO-AEG-1-Mut. A dual luciferase reporter assay was carried out in HCT-116 control cells. The cells were transfected with the recombinant plasmid and co-transfected with plasmid and miR-122 mimic. The luciferase activity was normalized with renilla luciferase activity. The findings revealed that the luciferase activity of wt-3′UTR-AEG-1 was decreased in the presence of miR-122, whereas the miR-122 mimic did not significantly influence the mut-3′-UTR-AEG-1 (Figure 3B). Thus, the dual luciferase assay system confirms the interplay between AEG-1 and miR-122.

### 3.5. AEG-1/MTDH protein was downregulated in miR-122 overexpressed colon cancer cells

Immunofluorescence staining was performed in the control and miR-122 transfected cell lines to assess the AEG-1/MTDH protein expression. Consistent with the gene expression analysis, the immunofluorescence analysis showed that the AEG-1/MTDH protein expression was downregulated in miR-122 overexpressed cells. The primary cell lines SW480 and HCT-116 had a higher response for the miR-122 transfection when compared to the metastatic cell line SW620 (Figure 3C).

### 3.6. TCGA-COAD data analysis to find out the clinical significance of miR-122 in colon cancer patients

**Figure 4.**
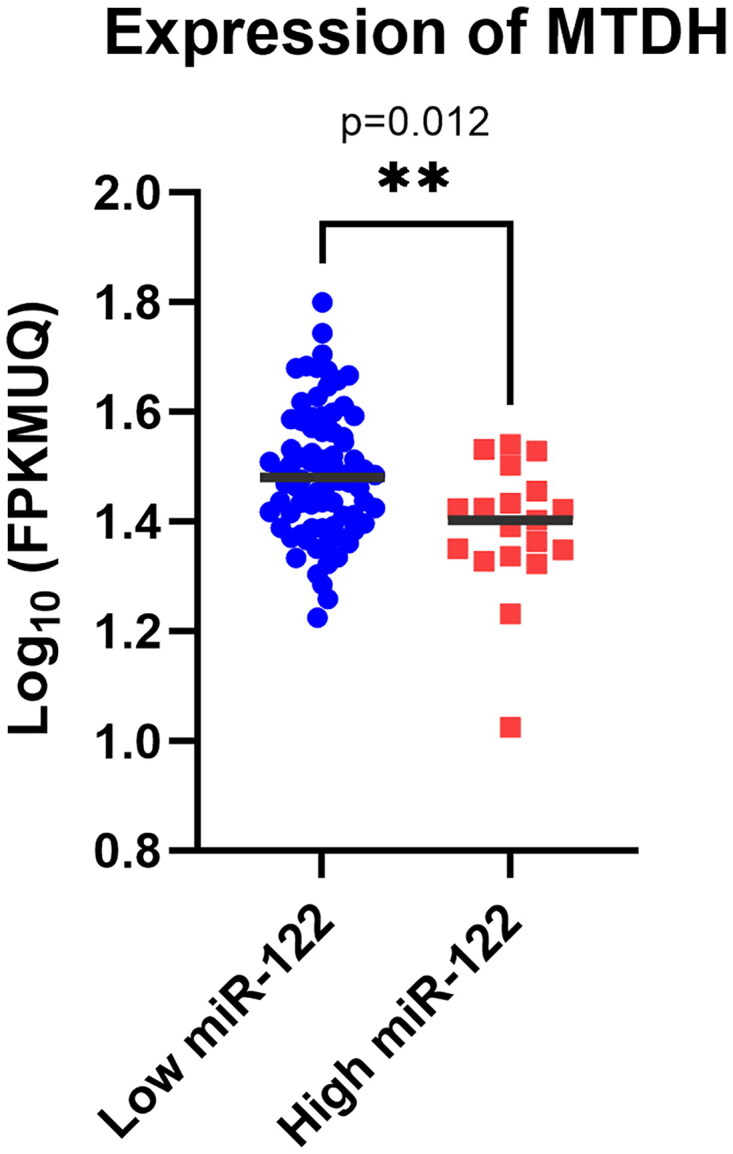
Expression analysis of AEG-1/MTDH in low and high expression of miR-122.

The data of colon cancer patients were obtained from the TCGA-COAD database. Among them, 42% were male patients, and 56.6% were female patients. Cancer patients had a median age of 68. The histological type of all tumours was colon adenocarcinoma .The TNM tumour stages included 127 patients diagnosed with ‘stage I’ (12.5%), ‘stage II’ (43%), ‘stage III’ (29%) and ‘stage IV’ (10%) (Figure 4). The whole data were distributed into two groups with miR-122 median value. Lower miR-122 expression had higher AEG-1/MTDH expression (*p =* 0.012) and *vice versa*.

### 3.7. Overexpression of miR-122 in AOM/DSS-induced colon tumor mouse model

**Figure 5.**
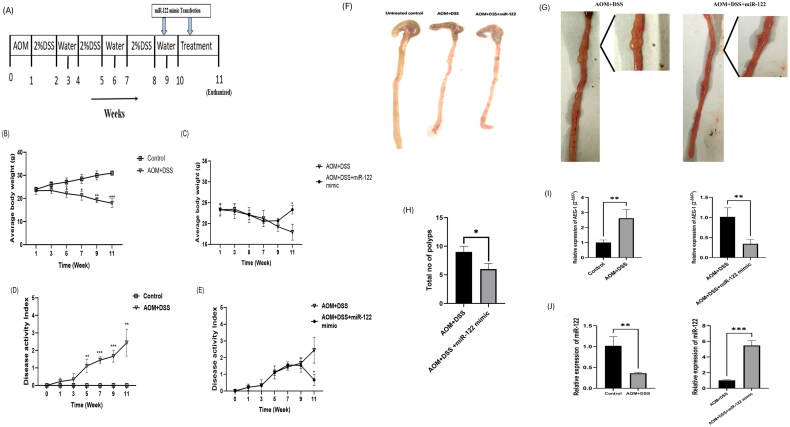
(A) Timeline for treatment of mice. (B,C) Average body weight of mice groups. (D,E) Disease activity index of different mice groups. (F) Gross morphology of control, AOM/DSS, AOM/DSS+miR-122 treated mouse colon tissue. (G) Magnified morphological view of AOM/DSS and AOM/DSS+miR-122 treated mouse colon tissue. (H) Total number of polyps found in AOM/DSS (*n* = 4), AOM/DSS+miR-122 mimic treated group (*n* = 4). (I) Relative expression of *AEG-1/MTDH* gene in colon tissue isolated from different groups. (J) Relative expression of miR-122 in colon tissue isolated from different groups.

The tumour was induced using AOM/DSS treatment, and the timeline for the induction of the tumour is shown in Figure 5A. The average loss of weight, feces consistency, and signs of bleeding in the rectal area were recorded daily to analyze the DAI. The mice that showed excessive weight loss and rectal bleeding were euthanized immediately. The average body weight loss was significantly observed from the 5^th^ week of tumour induction in the AOM/DSS group, and there was continuous weight loss till the 11^th^ week. However, in the miR-122 mimic treatment group, the gain of weight was seen at week 11 (*p* = 0.0125) (Figures 5B,C). The DAI was found to be relatively higher in the AOM/DSS mice group than in the control and miR-122 transfected groups (Figures 5D,E). The DAI significantly increased from the 5^th^ week to the 11^th^ week in the AOM/DSS group. The miR-122 mimic-treated group had low DAI in the 11^th^ week (*p* = 0.0214).

Figure 5F shows the gross morphology of the colon isolated from the control group, AOM/DSS group, and AOM/DSS+miR-122 mimic treated group, whereas Figure 5G shows the magnified view of polyps present in the AOM/DSS and AOM/DSS+miR-122 mimic treated groups. The results suggest that miR-122 treatment may help to control inflammation and disease progression in the mice. As the result of inflammation caused by AOM/DSS, spleen enlargement was observed in the tumour-induced group. The inflammation and polyp size were reduced in the miR-122 mimic transfected group. Similarly, the number of polyps was higher in the AOM/DSS group than in the AOM/DSS+miR-122 group (Figure 5H). Quantitative real-time RT-PCR was carried out to assess the expression of miR-122 and AEG-1/MTDH in the control, AOM/DSS, and miR-122 treated groups. The results imply that there was a substantial reduction of *miR-122* expression (*p* = 0.007) and increased expression of *AEG-1/MTDH* (*p* = 0.009) in the AOM+DSS group. However, in the AOM/DSS+miR-122 treated group, *AEG-1/MTDH* expression was reduced (*p* = 0.0095), and *miR-122* expression was increased to few folds (*p* = 0.0003) (Figures 5I,J).

### 3.8. Histopathology and immunohistochemistry of tissue sections

**Figure 6.**
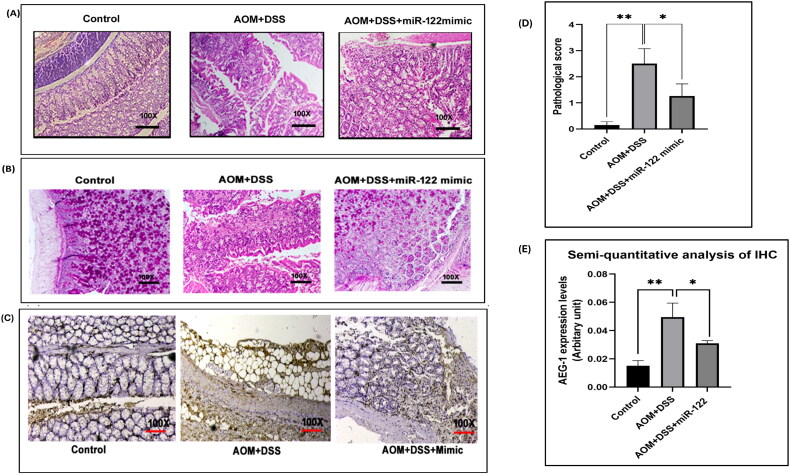
(A) H&E staining in formalin-fixed mouse colon tissue. (B) Periodic Acid Schiff staining in formalin-fixed mouse colon tissue. (C) Immunohistochemical staining of AEG-1/MTDH protein. (D) Pathological scoring in formalin-fixed mouse colon tissue. (E) Semi-quantitative analysis of IHC of AEG-1/MTDH protein.

The colon tissues collected from the different mice groups were fixed with formalin and further processed for immunohistochemistry and histopathological analysis. Hematoxylin and Eosin (H&E) staining revealed that the control group showed normal crypts, whereas the AOM/DSS mice group showed an increase in inflammatory cells, severe crypt distortion, and mucin depletion. MiR-122 mimic-treated mice group showed moderate crypt distortion, mucin depletion and inflammatory cells (Figure 6A). Scoring of histology slides was done, and Image-J software was used for analysis. The histopathological score was calculated by summing the scores of epithelial damage, crypts, and mononuclear cell infiltration. The pathological score was very high in the AOM/DSS group in contrast to the miR-122 treated group (Figure 6D). Similarly, Periodic Acid Schiff (PAS) staining was done to observe the intact goblet cells. The AOM/DSS mice group had severely distorted crypts and mucosa, whereas miR-122 treatment reduced the severity of distortion (Figure 6B).

Immunohistochemical staining and semi-quantitative analysis were performed to quantify the AEG-1/MTDH protein expression in the formalin-fixed tissue sections (Figure 6C). The images were captured and semi-quantified with Image-J software. The expression level was represented in an arbitrary unit. The semi-quantitative analysis showed that AEG-1/MTDH expression was relatively high in the AOM/DSS group in contrast to the control (*p* ≤ 0.005) and AOM/DSS+miR-122 (*p* ≤ 0.03) groups. Our results suggest that miR-122 overexpression modulates the inflammation in the colon and may suppress the tumor progression (Figure 6E).

## 4. Discussion

It has been well established that miRNAs play an important role in cellular development, and dysregulated miRNA expression contributes to cancer development and progression [12]. The role of miRNAs in the innate and adaptive immune systems development and operation is becoming more well-acknowledged, and many immune-related disorders have been linked to alterations in the miRNA expression profile [13–15]. Nevertheless, the role of *miR-*122 in colon tumour progression is unexplored. We used miRNA PCR array-based profiling and functional enrichment using miRnet as part of the screening step. Functional enrichment allowed us to prioritize the gene and uncover its connections to disease progression. The expression profiling of miRNAs in the HCT-116 colon cancer cell line revealed that hsa-*miR-21-5p*, *hsa-miR-222-3p*, and *hsa-miR-29a-3p* are highly expressed. *MiR-21-5p* is associated with lymph node metastasis and tumour malignancy, and has a negative impact on patient survival [16].

Studies have shown that *miR-222* promotes cell proliferation, migration, and invasion of colorectal cancer cells [17,18]. Stage II colorectal cancer samples have been found to have higher *miR-29a* expression and were found to be upregulated in colon tumour tissue obtained from colorectal liver metastasis (CRLM) patients [19]. The expression of miRNAs, namely *hsa-miR-133b*, *hsa-miR-155-5p*, *hsa-miR-34c-5p*, and*miR-146a-5p*, was significantly downregulated in HCT-116 cells and was found to act as tumour suppressor miRNAs [20–23]. The elucidation of other downregulated miRNAs, such as *has-miR-184*, *hsa-miR-1-3p*, and *hsa-miR-122-5p* has been carried out in HCC [24–26], however, there are only few studies in colorectal cancer.

Comprehensive screening and functional enrichment analysis led to the selection of *miR-122* as it was an ideal candidate due to its tumour suppressive properties and it also plays an important role in the regulation of angiogenesis, cell cycle, and apoptosis. The primary reason for selecting miRNA-122 for further investigation is due to the well-established fact that 50% of individuals with colorectal cancer develop liver metastases, either synchronously or metachronously. Although the abnormal expression of miR-122 has been extensively studied in HCC, its role in colorectal cancer with liver metastasis warrants deeper exploration [27,28]. MiRNAs play a crucial role in regulating the expression of numerous oncogenes and tumour suppressor genes, and its dysregulation contribute to cancer pathogenesis [29]. The loss of control over oncogenes by the downregulated miRNAs, such as miR-122 is another compelling reason to explore it, as this could shed light on the underlying processes in colorectal cancer. Our previous study showed that transfection of colon cancer cell lines with *miRNA-122* not only inhibited the proliferation and migration of colon cancer cells but also induced programmed cell death [9]. Our current study proved that overexpression of *miRNA-122* inhibited the escape of colon cancer cells from immune surveillance. The survival of cancer cells is highly influenced by key growth factors and pro-inflammatory cytokines, namely VEGF and IL-6 [30,31]. These factors often help the cancer cells to escape from the host immune system [13,29,32,33]. Studies have reported that IL-6 secretion might activate the STAT-3 pathway and consequent stimulation of the NF-κB pathway [34]. Similarly, VEGF induces the angiogenesis process to promote tumour growth and metastasis. VEGF levels are high in colon cancer and are correlated with the development of the disease [31]. Cytokine quantification by ELISA confirmed that overexpression of miR-122 in colon cancer cells reduced the levels of inflammatory cytokines IL-6 and VEGF. Cancer cells lack apoptosis as an elimination mechanism of the damaged cells, which is a major concern in anti-cancer treatment. Apoptosis is characterized by the proteolysis of several intracellular proteins, which are cleaved either directly or indirectly by caspase activity and the release of cytochrome C from mitochondria, which is regulated by BCL2. This cytochrome C activates *Caspase-9*, which activates *Caspase-3* [35,36]. *BAX,* pro-apoptotic protein deregulation is strongly associated with the progression of colon cancer [37]. In this context, our present study demonstrates that overexpression of miR-122 in colon cancer cells lowered the levels of pro-inflammatory cytokines, and, interestingly, it elevated the expression of mitochondrial multifunctional protein *cytochrome C*, *BAX, Caspase-9* and *Caspase−3*, and decreased the *BCL2* expression.

Although miRNAs have multiple targets, their function in the regulation of tumorigenesis depends on the few targets [38]. Bioinformatics tools like miRwalk and target scan were employed to elucidate the underlying mechanisms and find the putative target of miR-122. Of all the predicted genes, *AEG-1/MTDH* were taken for further validation by dual luciferase assay as it plays key roles in tumorigenesis in various cancers. AEG-1/MTDH and lysine-rich CEACAM1 co-isolated protein (*LYRIC*), an oncogene well-known are increased in many cancers [39]. Additionally, it is linked to an aggressive metastatic stage and has an impact on the prognosis and the survival of cancer patients [39,40]. *AEG-1/MTDH* promotes cell survival, proliferation, migration, and invasion through its involvement in several signalling pathways. Studies have shown that upregulation of *AEG-1/MTDH* has been associated with increased expression of VEGF and other epithelial-mesenchymal transition (EMT) markers [41,42]. Our study pointed out that elevated expression of AEG-1/MTDH was associated with lower levels of miRNA-122 and *vice versa*.

Zhang et al. [43] reported that miRNA-122 regulates Caspase-8 in mouse cardiomyocytes. Though they have reported the regulation, the interaction between them was still unknown as miR-122 does not have a direct binding sequence for caspase-8 [43]. So, considering the other intriguing factors, we suggest that miR-122 may regulate caspase-8 through binding with AEG-1/MTDH. Zhang et al. [44] studied the role of AEG-1/MTDH in regulating the apoptotic pathways. They suggested that *AEG-1/MTDH* depletion activates the caspase-8 and further apoptosis signals and *vice versa* [44]. Collectively, our results prove that* miR-122* downregulates the *AEG-1/MTDH* expression, and we speculate that it, in turn, may activate the apoptotic pathway for programmed cell death.

The AOM/DSS model is frequently utilized in research on colon tumorigenesis because it is cost-effective and highly reproducible. Studies employing this model have defined the processes of inflammation-related colon carcinogenesis in the gut and highlighted the significance of the inflammatory process in the development of colorectal cancer [45]. This model induces tumours with histological and molecular changes that closely mimic those seen in human colitis-associated cancer (CAC), making it a highly valuable tool for studying oncogenesis and chemoprevention in this disease [46]. Therefore, we adopted this model. miR-122 can function as a pro-apoptotic factor to drive tumour cell apoptosis and regulate tumour invasion [28]. In line with this, our results showed that miR-122 overexpression partially ameliorated the symptoms related to colonic inflammation in mice. The AOM/DSS tumour-induced mice group significantly lost weight over weeks when compared to the control and miR-122 mimic-treated group. Similarly, there was a substantial difference in the number of polyps between the AOM/DSS tumour-induced mice group and the miR-122 mimic-treated group. Previously, Tsai et al. [46] demonstrated the tumor-suppressing properties of miR-122 in HCC. They reported that restoration of miR-122 in HCC cell lines reduced EMT and cell invasion. They implanted the HCC cell line infected with miR-122 lentivirus in nude mice. The restoration of miR-122 reduced the overall tumour growth in this nude mouse model [46]. Moreover, miR-122 has many targets that are involved in important cancer signalling pathways, and it has a distinctive therapeutic significance in cancer. Taken together, our study, for the first time, establishes an association between AEG-1/MTDH, miR-122 and colon cancer. The expressions of miR-122 and AEG-1/MTDH, are Inversely correlated in colon cancer cells, demonstrating the critical role of miR-122 and its therapeutic potential in colon cancer. Further studies are needed to evaluate the miRNA mimic-based combinational therapy and specific drug delivery system.

## 5. Conclusions

Our study demonstrates that miRNA-122 expression is decreased in human colon cancer and its overexpression reduces the colon cancer progression in AOM/DSS induced tumor mouse model by regulating the AEG-1/MTDH expression, specifying its potential as a therapeutic target for colon cancer. Further research is required to validate the findings.

## Supplementary Material

Supplementary materials .pdf

## Data Availability

Data will be available from the corresponding author upon request.
